# Molecular Identification of Carnosine N-Methyltransferase as Chicken Histamine N-Methyltransferase-Like Protein (HNMT-Like)

**DOI:** 10.1371/journal.pone.0064805

**Published:** 2013-05-21

**Authors:** Jakub Drozak, Lukasz Chrobok, Olga Poleszak, Adam K. Jagielski, Rafal Derlacz

**Affiliations:** Department of Metabolic Regulation, Institute of Biochemistry, Faculty of Biology, University of Warsaw, Warsaw, Poland; Universität Stuttgart, Germany

## Abstract

Anserine (beta-alanyl-N(Pi)-methyl-L-histidine), a naturally occurring derivative of carnosine (beta-alanyl-L-histidine), is an abundant constituent of skeletal muscles and brain of many vertebrates. Although it has long been proposed to serve as a proton buffer, radicals scavenger and transglycating agent, its physiological function remains obscure. The formation of anserine is catalyzed by carnosine N-methyltransferase which exhibits unknown molecular identity. In the present investigation, we have purified carnosine N-methyltransferase from chicken pectoral muscle about 640-fold until three major polypeptides of about 23, 26 and 37 kDa coeluting with the enzyme were identified in the preparation. Mass spectrometry analysis of these polypeptides resulted in an identification of histamine N-methyltransferase-like (HNMT-like) protein as the only meaningful candidate. Analysis of GenBank database records indicated that the *hnmt-like* gene might be a paralogue of histamine N-methyltransferase gene, while comparison of their protein sequences suggested that HNMT-like protein might have acquired a new activity. Chicken HNMT-like protein was expressed in COS-7 cells, purified to homogeneity, and shown to catalyze the formation of anserine as confirmed by both chromatographic and mass spectrometry analysis. Both specificity and kinetic studies carried out on the native and recombinant enzyme were in agreement with published data. Particularly, several compounds structurally related to carnosine, including histamine and L-histidine, were tested as potential substrates for the enzyme, and carnosine was the only methyl group acceptor. The identification of the gene encoding carnosine N-methyltransferase might be beneficial for estimation of the biological functions of anserine.

## Introduction

Anserine (β-alanyl-N-π-methyl-L-histidine) and balenine (β-alanyl-N-τ-methyl-L-histidine) are naturally occurring derivatives of carnosine (β-alanyl-L-histidine) that have been reported to be present in skeletal muscle and the central nervous system of vertebrates [1]. In contrast to carnosine that is present at high concentrations (in the range of 0.6 to 30 mM) in excitable tissues of almost all vertebrates, including human beings [2], [3], the occurrence of anserine and balenine is more peculiar. Balenine has been found exclusively in snake muscles and marine mammals such as whales and dolphins (up to 45 mM), while anserine was reported to be a major L-histidine-containing dipeptide in avian tissues (up to 43 mM in chicken pectoral muscle) [3]. However, it has also been detected in muscle of fish (2.5 up to 41 mM), cats (8 mM) and rabbits (17 mM), but not in frogs and humans [1], [2].

Because of a limited presence of balenine in vertebrates, much effort has been put to understand the physiological role of both carnosine and anserine. Originally, these two dipeptides have been postulated to serve as buffers neutralizing lactic acid produced in working muscle due to their abundance and p*Ka* which is close to the physiological pH [4]. However, this notion has not provided any explanation for the synthesis of anserine which shows buffer capacity similar to that of carnosine. Recently, histidine-containing dipeptides have been considered to exert a more complex effect on cell and tissue metabolism *via* their potent antiglycemic [5], [6], antiglycation [7] and antioxidant properties [8]. Unfortunately, no definitive explanation of their physiological importance has been provided.

Information on the enzymes that catalyze the formation of histidine-containing dipeptides have long been highly deficient. Recently, carnosine synthase has been identified as ATP-grasp domain-containing protein 1 and characterized biochemically, providing a new insight into the biosynthesis of carnosine [9]. On the other hand, very little is known about carnosine N-methyltransferase that catalyzes the synthesis of anserine. The enzyme has been only partially purified from various sources [10], [11] and shown to catalyze the transfer of methyl group of S-adenosyl-L-methionine to carnosine with a high substrate specificity, yielding anserine.

Anserine and other histidine-containing dipeptides are subjects of degradation by two proteins encoded by different genes in vertebrates [12]. The first one (CNDP2, EC 3.4.13.18) is a Mn^+2^-dependent cytosolic enzyme ubiquitously expressed in various tissues. This enzyme exhibits a broad specificity toward various dipeptides, and therefore it is named a “cytosolic non-specific dipeptidase”. The second one (CN1, EC 3.4.13.20) is a true carnosinase which catalyzes hydrolysis of carnosine and anserine and is found in serum and brain. Interestingly, these two forms of carnosinases are characterized by a much higher activity toward carnosine compared with anserine, suggesting that anserine is a more metabolically stable derivative of carnosine [13].

The occurrence of anserine in excitable tissues is highly variable among vertebrates, making it difficult to provide a definitive interpretation of its physiological function. Thus, further progress on the role of anserine might benefit from the identification of the enzyme that synthesizes this compound. In the current investigation, carnosine N-methyltransferase was purified from chicken muscle, a rich source of the enzyme, characterized and identified using mass spectrometry analysis.

## Materials and Methods

### Ethics Statement

All animal use procedures were approved by the First Warsaw Local Commission for the Ethics of Experimentation on Animals (Permit Number: 157/2011). Pectoral muscles were taken from animals euthanized with an overdose of sodium pentobarbital, and all efforts were made to minimize suffering.

### Chemicals and reagents

Reagents, of analytical grade whenever possible, were from Sigma-Aldrich (Poznan, Poland), Roche Diagnostics (Warsaw, Poland) or Merck (Darmstadt, Germany). S-(methyl-^3^H)adenosyl-L-methionine was purchased from either American Radiolabeled Chemicals (St. Louis, USA) or PerkinElmer (Boston, USA). DEAE-Sepharose, Q-Sepharose, Superdex 200, Superdex 75 resins, 1 ml HisTrap HP (Ni^2^+ form) and PD-10 columns were obtained from GE Healthcare Bio-Sciences (Uppsala, Sweden). AG50W-X4 (200 mesh) resin came from Sigma-Aldrich (Poznan, Poland) and Vivaspin-20 centrifugal concentrators were from Sartorius Stedim (Kostrzyn, Poland). Recombinant human histamine N-methyltransferase was purchased from ProSpec (Ness Ziona, Israel). All other enzymes and DNA modifying enzymes as well as the TurboFect transfection reagent were obtained from Fermentas (St-Leon-Rot, Germany).

### Assay of Carnosine N-Methyltransferase Activity

Carnosine N-methytransferase activity was determined by measuring the incorporation of (^3^H)methyl group from S-(methyl-^3^H)adenosyl-L-methionine ((^3^H)SAM) into carnosine. The standard incubation mixture (0.11 ml) contained 50 mM Hepes, pH 7.5, 10 mM KCl, 1 mM EGTA, 1 mM MgCl_2_, 1 mM DTT, 20 mM carnosine (or other acceptors) and 1 µM (^1^H+^3^H)SAM (from about 400×10^3^ to 700×10^3^ cpm). Blanks containing no carnosine were included in all assays. The reaction was started by the addition of enzyme preparation and carried out at 37°C for 20 min unless otherwise described. Anserine production was linear for at least 30 min under all conditions studied. The incubation was stopped by the addition of 0.1 ml of the reaction medium to 0.2 ml of ice-cold 10% (w/v) HClO_4_. The samples were diluted with 0.12 ml of H_2_O and centrifuged at 13,000× g for 10 min. After neutralization of the supernatant with 3 M K_2_CO_3_, the salts were removed by centrifugation (13,000× g for 10 min) and the clear supernatant was diluted 5 times with 20 mM Hepes, pH 7.5 and 2 ml were applied to AG50W-X4 columns (1 ml, Na^+^ form), equilibrated with 20 mM Hepes, pH 7.5. The columns were washed with 5×2 ml of 20 mM Hepes pH 7.5 to remove minor radioactive contaminants of the radio reagent or to elute methylated derivatives of imidazole 4-acetic acid or imidazole 4-acrylic acid (urocanic acid). Anserine and methylated forms of L-histidine and imidazole were eluted with 5×2 ml of 20 mM Hepes pH 7.5 containing 0.5 M NaCl. To elute a non-consumed (^1^H+^3^H)SAM, the columns were washed with 4×2 ml of 1 M NH_4_OH.

Since the above described fractionation procedure does not separate (^1^H+^3^H)N-methylhistamine from (^1^H+^3^H)SAM, a slightly modified method of Bowsher and coworkers [14] was applied to measure histamine methylation in the presence of carnosine N-methyltransferase. Briefly, the reaction was initiated by the addition of enzyme preparation to the reaction mixture (0.11 ml) containing 50 mM Hepes, pH 7.5, 10 mM KCl, 1 mM EGTA, 1 mM MgCl_2_, 1 mM DTT, histamine at 10 µM up to 10 mM concentration and 1 µM (^1^H+^3^H) SAM (about 400×10^3^ cpm) and carried out at 37°C for 10 min. Under these conditions with 1.3 microunit of human histamine N-methytransferase, the assay was linear up 20 min. Histamine methylation in the presence of human histamine N-methytransferase was followed in every assay serving as a positive control. The incubation was stopped by the addition of 0.1 ml of the reaction mixture to 0.2 ml of ice-cold 0.2 M sodium borate, pH 11.0, followed by the addition of 1.3 ml of toluen/isoamyl alcohol (3∶2). After vortexing for 20 min and centrifugation at 1500× g, 1 ml of the organic phase was transferred to 0.3 ml of 0.5 M HCl. The samples were vortexed for 20 min, centrifuged and the organic phases were removed by aspiration. A 250 µl aliquot of the aqueous phase was then transferred to a scintillation vial.

In all cases, the samples to be counted were mixed with 6 volumes of scintillation fluid (Ultima Gold, Perkin-Elmer) and the incorporated radioactivity was analyzed with a Beckman LS6000 IC liquid scintillation counter.

### Purification of Chicken Carnosine N-Methyltransferase

Chicken pectoral muscle (130 g) was homogenized with 4 volumes (w/v) of buffer A (50 mM Hepes, pH 7.6, 10 mM KCl, 1 mM DTT, 1 mM EGTA, 1 mM MgCl_2_, 5 µg/ml leupeptin and 5 µg/ml antipain) with a Waring Blender 7011HS. The homogenate was centrifuged for 40 min at 20,000× g. The supernatant (470 ml) was applied to a DEAE-Sepharose column (200 ml) equilibrated with buffer A. The column was washed with 600 ml of buffer A, developed with a NaCl gradient (0–0.5 M in 1000 ml) in buffer A and fractions (7 ml) were collected. The most active fractions of the DEAE column (56 ml) were diluted to 220 ml with buffer B (50 mM Tris HCl, pH 8.0, 10 mM KCl, 1 mM DTT, 1 mM EGTA, 1 mM MgCl_2_, 5 µg/ml leupeptin and 5 µg/ml antipain) and applied to a Q-Sepharose column (45 ml) equilibrated with buffer B. The column was washed with 135 ml of buffer B containing 25 mM NaCl, and the retained protein was eluted with a NaCl gradient (25–500 mM in 350 ml in buffer B). The most active fractions (15 ml) were pooled, concentrated to 2.6 ml in Vivaspin-20 ultrafiltration devices and loaded on a Superdex 200 16/60 column (120 ml) equilibrated with buffer A containing 100 mM NaCl. To obtain more purified enzyme preparation for kinetic studies and tandem mass spectrometry, the most active fractions (4 ml) of Superdex 200 purification step were pooled again, concentrated to 1 ml in Vivaspin-20 ultrafiltration devices and fractionated on a Superdex-75 10/300 GL column (24 ml) equilibrated with buffer A containing 100 mM NaCl. All purification steps were performed at 4°C and the enzymatic preparation was stored at −70°C between steps. Protein concentration was determined spectrophotometrically according to Bradford [15] using bovine γ-globulin as a standard. Protein content in the polyacrylamide-SDS gel bands, which co-eluted with carnosine N-methyltransferase activity in the last purification step (Superdex-75), was quantitated by densitometric analysis using ImageJ software (NIH, USA).

### Identification of Chicken Carnosine N-Methyltransferase by Tandem Mass Spectrometry

The bands co-eluting with carnosine N-methyltransferase activity in the Superdex 75 purification step were cut from a 10% polyacrylamide-SDS gel and digested with trypsin. In-gel digestions of the peptides were performed as described in [16]. To identify all proteins present in the most active enzyme fraction from the Superdex 75 purification step, 10 µg protein from the fraction 17^th^ was reduced by incubation at 50°C for 30 min in the reaction mixture containing 100 mM NH_4_HCO_3_, 0.1% RapiGest (Waters, USA) and 5 mM DTT. Then the reaction mixture was supplemented with 9 mM iodoacetamide to alkylate proteins. Following 40 min alkylation, 2 µg proteomics grade porcine trypsin was added to the mixture and the proteins were digested overnight at 30°C. Peptides were analyzed by nanoUPLC-tandem mass spectrometry employing Acquity nanoUPLC coupled with Synapt G2 HDMS Q-TOF mass spectrometer (Waters, USA) fitted with a nanospray source and working in MS∧E mode under default parameters. Briefly, products of protein digestion (1 µg) were loaded onto a Waters Symmetry C18 trapping column (20 mm×180 µm) coupled to the Waters BEH130 C18 UPLC column (250 mm×75 µm). The peptides were eluted from these columns in a 1–85% gradient of acetonitrile in water (both containing 0.1% formic acid) at a flow rate of 0.3 µl/min. The peptides were directly eluted into the mass spectrometer. Data were acquired and analyzed using MassLynx 4.1 software (Waters, USA) and ProteinLynx Global Server 2.4 software (Waters, USA) with a False Discovery Rate ≤4%, respectively. To identify chicken histamine N-methyltransferase-like protein, all chicken protein sequences available in NCBI protein database were downloaded, randomized and used as a databank of the MS/MS software.

### Overexpression and Purification of Chicken Recombinant Histamine N-Methyltransferase-like Protein

Chicken total muscle RNA was prepared from 200 mg of pectoral muscle with the use of TriPure reagent (Roche) according to the manufacturer's instructions. Muscle cDNA was synthesized using Moloney murine leukemia virus-reverse transcriptase (Fermentas, St-Leon-Rot, Germany), with oligo(dT)_18_ primer and 2.5 µg of total RNA according to the manufacturer's instructions.

Chicken muscle cDNA was used to PCR-amplify the open reading frame encoding histamine N-methyltransferase-like protein (HNMT-like, GenBank Accession Number XM_001234739.1) using Pfu DNA polymerase in the presence of 1 M betaine. HNMT-like was amplified using a 5′ primer containing the initiator codon (GTGGAATTCGCCACCATGGAGCCCACCCCTGAGAT) preceded by the Kozak consensus sequence [17] and an EcoRI site and a 3′ primer (CAGTCTAGAATAGGATTCCGCTACGATCATAC) in which the original stop codon was replaced by ATA codon flanked by a XbaI site. The amplified DNA product of the expected size was digested with the appropriate restriction enzymes and cloned into the pEF6/Myc-His A expression vector (Invitrogen, USA), which allows the production of proteins with an C-terminal His6 tag, and verified by sequencing. For transfections, COS-7 cells (CLS Cell Lines Service GmbH, Germany) or HEK-293T (a kind gift of Dr. Maria Veiga-da-Cunha, de Duve Institute, Brussels) were plated in 100-mm Petri dishes at a cell density of 1.7×10^6^ or 2.1×10^6^ cells per plate, respectively, in Dulbecco's minimal essential medium supplemented with 100 units/ml penicillin, 100 µg/ml streptomycin, and 10% (v/v) fetal bovine serum, and grown in a humidified incubator under 95% air and 5% CO_2_ atmosphere at 37°C. After 24 h, each plate was transfected with 5 µg of either unmodified pEF6/Myc-His A vector or the same vector encoding HNMT-like protein using the TurboFect transfection reagent according to the protocol provided by the manufacturer. After 48 h the culture medium was removed, the cells were washed with 5 ml phosphate buffered saline and harvested in 1 ml of 50 mM Hepes, pH 7.5, containing 10 mM KCl, 1 mM MgCl_2_, 1 mM EGTA, 5 µg/ml leupeptin and 5 µg/ml antipain. The cells were lysed by freezing in liquid nitrogen and after thawing and vortexing, the extracts were centrifuged at 4°C (20,000× g for 30 min) to remove insoluble material.

**Figure 1:**
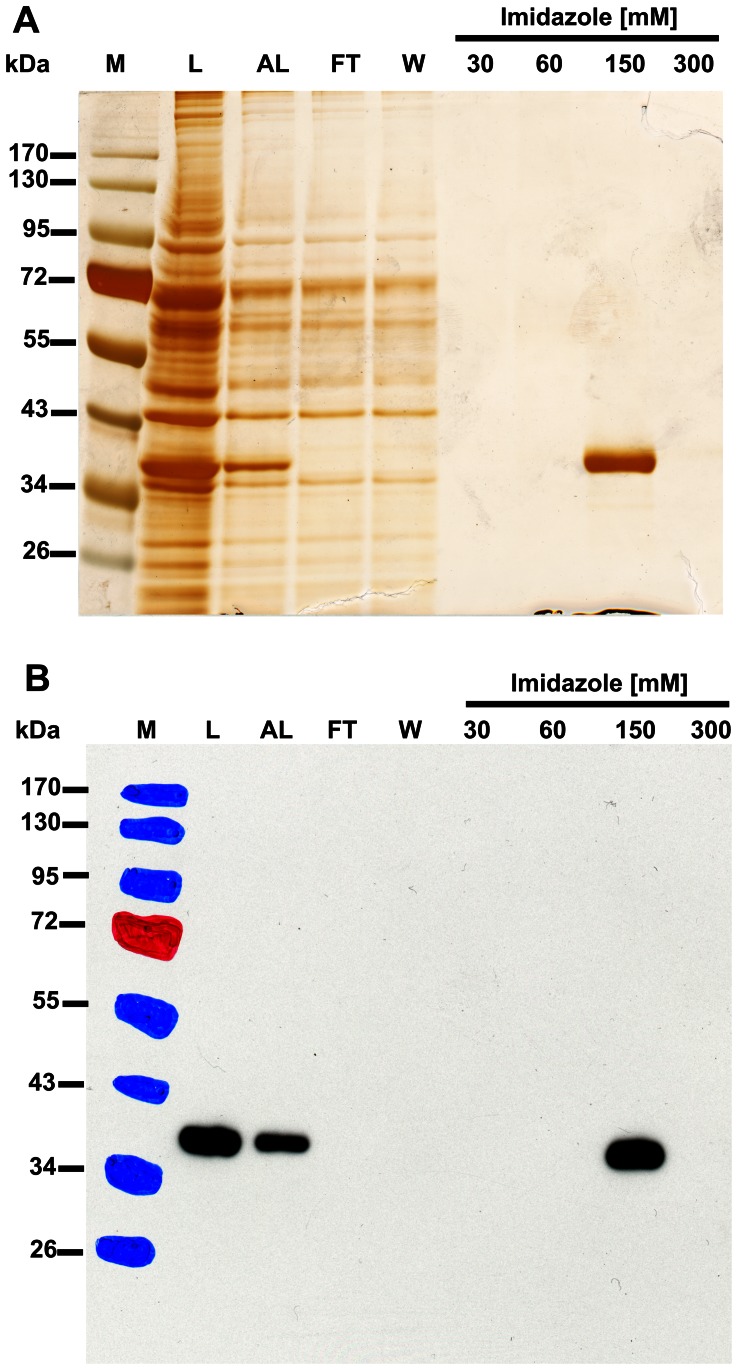
SDS-PAGE and Western-blot analysis of fractions obtained during purification of recombinant HNMT-like protein. Chicken HNMT-like protein was produced in COS-7 cells and purified to homogeneity by affinity chromatography on nickel-sepharose (HisTrap HP) as described under “Materials and Methods”. For the SDS-PAGE analysis (A), 20 µl of reduced sample from each fraction was loaded onto a 10% gel, electrophoresed and the resulting gel was then stained with silver [26]. For the Western-blot analysis (B), 1 µl of reduced sample from each fraction was loaded onto a 10% gel, electrophoresed and blotted to nitrocellulose membrane which was then sequentially probed with a mouse primary antibody against His6 tag and a horseradish peroxidase-conjugated goat anti-mouse antibody. Secondary antibody was detected through autoradiography using chemiluminescence. M, prestained protein marker; L, cell-free lysate of COS-7 cells overexpressing the recombinant enzyme; AL, 4-fold diluted lysate applied to the column; FT, flow through; W, wash; Fractions 30 to 300 were eluted with the indicated concentrations of imidazole. The pattern of prestained protein bands was copied from the blotting membrane onto ECL film using a set of felt-tip pens.

For the purification of chicken recombinant HNMT-like protein, the supernatant of COS-7 lysate (7 ml) was diluted 4-fold with buffer A (50 mM Hepes, pH 7.5, 300 mM NaCl, 10 mM KCl, 15 mM imidazole, 1 mM MgCl_2_, 5 µg/ml leupeptin and 5 µg/ml antipain) and applied on a HisTrap HP column (1 ml) equilibrated with the same buffer. The column was washed with 6 ml buffer A and the retained protein was eluted with a stepwise gradient of imidazole (5 ml of 30 mM, 5 ml of 60 mM, 7 ml of 150 mM and 5 ml of 300 mM) in buffer A. The recombinant protein was eluted with 150 mM imidazole in homogeneous form as confirmed by SDS-PAGE (Fig. 1). The enzyme preparation was desalted on PD-10 columns equilibrated with 50 mM Hepes, pH 7.5, 10 mM KCl, 1 mM DTT, 1 mM EGTA and 1 mM MgCl_2_. Protein concentration was determined spectrophotometrically according to Bradford [15] using bovine γ-globulin as a standard. The amount of 0.39 mg of pure recombinant enzyme was obtained from 56 mg of soluble COS-7 cell protein. The purified enzymes were supplemented with 2 mg/ml BSA and stored at −70°C.

### Western-blot analysis

Western blotting was performed according to the instructions provided by the manufacturer of ECL Plus Western Blotting Detection Reagent (GE Healthcare). Briefly, protein samples were resuspended in a reducing lane marker and sample buffer (Thermo), electrophoresed on a 10% Tris gel with Tris-glycine-SDS running buffer, then blotted to nitrocellulose membrane (Hybond-ECL, GE Healthcare) and sequentially probed with a mouse monoclonal primary antibody against His6 tag (IgG2a, GE Healthcare) and a horseradish peroxidase-conjugated goat anti-mouse secondary antibody (IgG, Sigma-Aldrich). Secondary antibody was detected through autoradiography (Hyperfilm ECL, GE Healthcare) using enhanced chemiluminescence (ECL Plus, GE Healthcare).

### Product Analysis

To obtain sufficient amount of the methylated dipeptide formed in the reaction catalyzed by recombinant chicken HNMT-like protein for mass spectrometry analysis, the reaction mixture was scaled up. Briefly, 3.3 µg chicken HNMT-like protein were incubated for 12 h at 37°C in 0.2 ml of a reaction mixture containing 25 mM Hepes, pH 7.5, 120 µg BSA derived from the enzyme preparation, 10 mM KCl, 1 mM EGTA, 1 mM MgCl_2_, 1 mM DTT, 3 mM carnosine in the absence or presence of 2 mM SAM. The reaction was stopped by the addition of 0.6 ml of acetonitrile. Precipitated protein was removed by centrifugation (13,000× g for 10 min) and the clear supernatants were analyzed by HPLC-Hydrophilic Interaction Chromatography (HPLC-HILIC) according to a slightly modified method of Mora and coworkers [18]. Briefly, carnosine and anserine were separated by the gradient mode on Supelco Ascentis Si column (2.1×250 mm, 5 µm) using Dionex ICS-3000 chromatograph (Thermo Scientific, USA). Mobile phases consisted of solvent A, containing 0.65 mM ammonium acetate, pH 5.5, in water/acetonitrile (25∶75), and solvent B, containing 4.55 mM ammonium acetate, pH 5.5, in water/acetonitrile (70∶30). The separation was performed in a linear gradient from 0 to 100% of solvent B for 13 min at flow rate 0.4 ml/min followed by the column wash in 100% of solvent B for 10 min and equilibration for 10 min under the initial conditions. The column eluate was monitored by UV detector at λ = 214 nm followed by a mass spectrometer. All mass spectral analysis were performed on Synapt G2 HDMS Q-TOF mass spectrometer fitted with an electrospray source (Waters, USA). The detector worked in positive MS/MS mode. The ESI-MS source was set at temperature 100°C, capillary voltage 3.5 kV and cone voltage 40 V. The flow-rate of the nebulizer gas (nitrogen) was 700 l/h. To confirm the structure of anserine precursor ion, collision-induced dissociation experiments were run by selecting the target ion (*m/z* 241). The trap collision energy was 20 eV.

### Calculations

Vmax , Km and kcat for the methyltransferase activity of studied enzymes were calculated with Prism 4.0 (GraphPad Software, USA) using a nonlinear regression.

## Results

### Purification and Identification of Chicken Carnosine N-Methyltransferase

**Figure 2:**
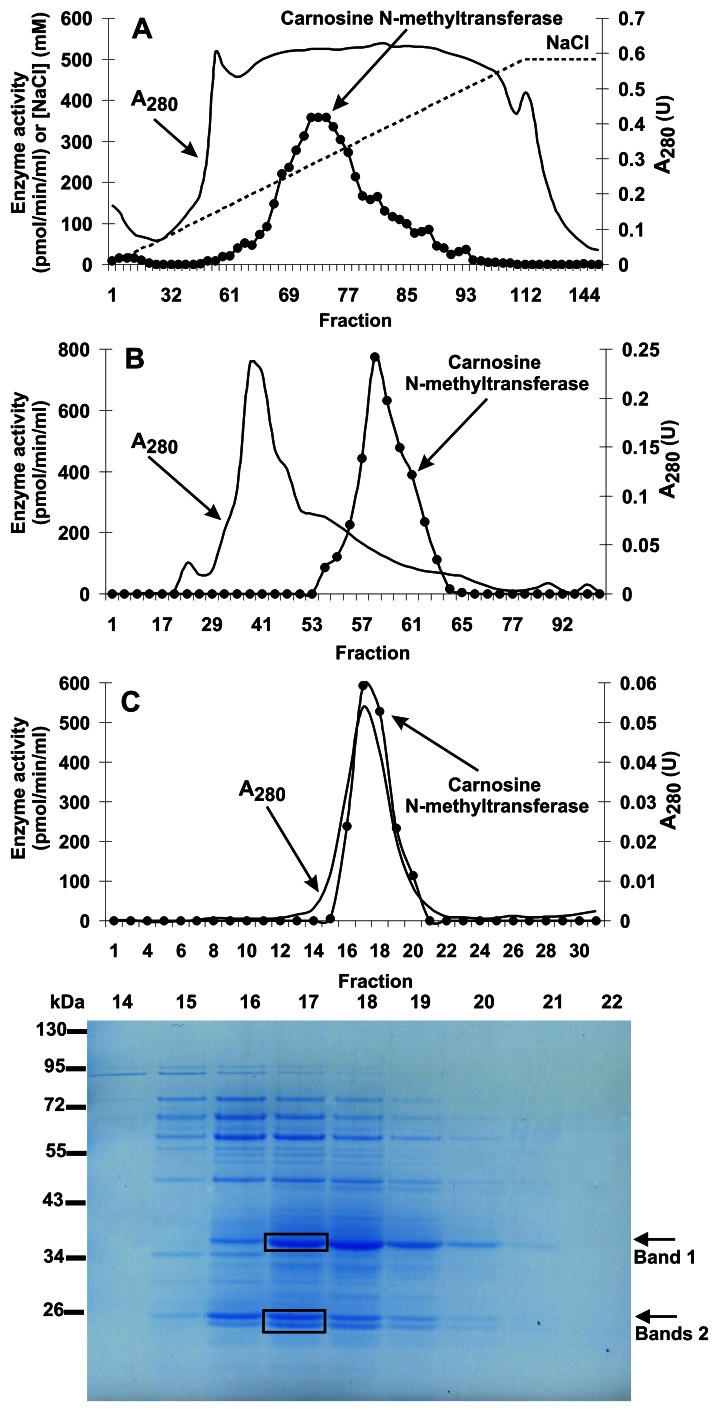
Purification of chicken carnosine N-methyltransferase. The enzyme was purified by chromatography on DEAE-Sepharose (A), Q-Sepharose (not shown), Superdex 200 (B), and Superdex 75 (C) as described in “Materials and Methods” section. Fractions were tested for carnosine N-methyltransferase activity. Protein concentration was determined in the most active fractions with the Bradford assay. The indicated fractions eluted from the Superdex 75 column were analyzed by SDS-PAGE and the gel was stained with Coomassie Blue. The indicated bands were cut out of the gel, submitted to trypsin digestion and analyzed by tandem mass spectrometry.

During its purification, carnosine N-methyltransferase activity was assayed by measuring the incorporation of (^3^H)methyl group of (^3^H)SAM into anserine. Carnosine N-methyltransferase was purified from chicken breast muscle about 640-fold by a procedure involving chromatography on DEAE-Sepharose, Q-Sepharose, Superdex 200, and Superdex 75. The methyltransferase was eluted as a single peak in each of the purification steps (Fig. 2), indicating the presence of a single enzyme species. The gel filtration step on Superdex-200 revealed that the molecular weight of the native carnosine N-methyltransferase was equal to about 45 kDa (not shown). The overall yield of the purification was only about 0.6% (Table 1) due to the fact that only the fractions exhibiting at least 75% activity of the most active one were used for the next step procedure.

**Table 1:** Purification of carnosine N-methyltransferase from chicken pectoral muscle.

Fraction	Volume (ml)	Total protein (mg)	Total activity (pmol min^−1^)	Specific activity (pmol min^−1^ mg^−1^)	Purification (-fold)	Yield (%)
20 000× *g* supernatant	470	14839	47485	3.2	1	100
DEAE Sepharose	56	622	18045	29.0	9	38.0
Q Sepharose	15	55	7227	135.3	42	15.2
Superdex 200	4	1.20	2274	1946.0	608	4.8
Superdex 75#	0.5	0.14	297	2053.0	642	0.6

# The data represent values for the most purified fraction.

**Figure 3:**
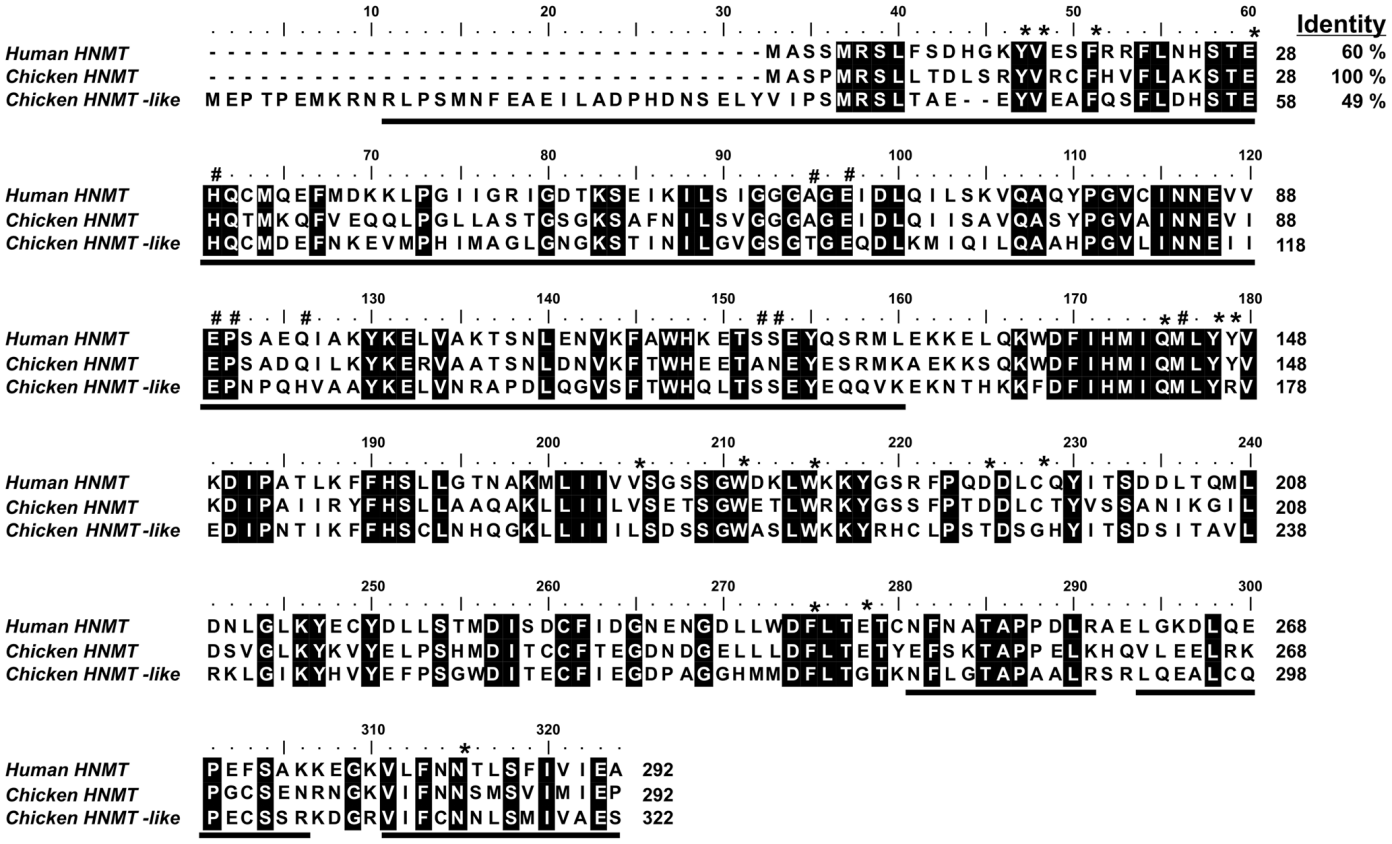
Amino acid sequence alignment of human HNMT with its chicken orthologue and chicken HNMT-like protein. Sequences were obtained with following GenBank accession numbers: human HNMT (NP_008826.1), chicken HNMT (XP_422143.2) and HNMT-like protein (XP_001234740.1). The chicken HNMT-like protein sequence has been confirmed by PCR amplification of the cDNA and sequencing. Percentage of amino acid identities with chicken HNMT is given in the upper right. Fully conserved residues are highlighted with a black background. Residues of human HNMT interacting with either SAM or histamine are marked by hashes or asterisks, respectively [19]. The peptides identified by mass spectrometry in the protein purified from chicken pectoral muscle are underlined in the chicken sequence.

Results of the SDS-PAGE analysis indicated that carnosine N-methyltransferase activity was coeluted with three major polypeptide bands of about 23–26 and 37 kDa in the last purification step (cf. Fig. 2). The bands were cut out from the gel, digested with trypsin, and the resulted peptides were analyzed by MS/MS and compared to the Uniprot reference proteome of chicken (downloaded on February 2012). The analysis indicated that the bands contained numerous proteins, but surprisingly, none of them appeared to be a methyltransferase (not shown). Since these negative results might have resulted from either a poor extraction of peptides from the gel or the absence of carnosine N-methyltransferase sequence in the Uniprot database, both the gel bands and the whole fraction 17^th^ of Superdex 75-purification step were reanalyzed by tandem mass spectrometry against a data bank containing all chicken protein sequences available at NCBI Protein database (presented in May 2012). This analysis revealed that HNMT-like protein was the only methyltransferase present in both the gel filtration fraction and the gel band 1 (Table 2). Ten matching peptides (underlined in Fig. 3) were found to cover about 58% of the sequence. This findings indicated that HNMT-like protein corresponded to the carnosine N-methyltransferase, even though, HNMT-like protein was not the most scored hit of the identification process, confirming the suggestion that further purification would have been required to obtain homogenous enzyme.

**Table 2:** Proteins identified in the fraction 17^th^ of Superdex 75 purification step and the gel bands submitted to trypsin digestion and MS/MS analysis.

Source of protein	Protein name	GenBank accession number	PLGS score	Coverage (*%*)
**Gel filtration fraction** *	WDR1 protein	AAD05042.1	28859	78
	Phosphoserine phosphatase	NP_001239201.1	25341	70
	Phosphoglycolate phosphatase	CAH65023.1	23691	79
	Myosin light chain 1	P02604.3	22148	89
	Rho GDP-dissociation inhibitor 2	XP_416182.1	21235	66
	Transforming protein RhoA	AAC18962.1	14618	50
	HD domain containing protein 2	XP_419755.1	14065	61
	Crk-like protein	XP_415233.1	13243	80
	Glucose-1,6-bisphosphate synthase	XP_001233129.2	12236	61
	Osteoglycin	AAD21085.1	11597	36
	RNH1 ribonuclease/angiogenin inhibitor 1	CAG32305.1	11440	75
	Mitogen activated protein kinase kinase 6	XP_003642396.1	11350	56
	Histamine N-methyltransferase like	XP_001234740.1	9103	58
**Gel Band 1**	Phosphoglycolate phosphatase	CAH65023.1	2129	70
	Protein FAM151B like	XP_003643128.1	1909	16
	Osteoglycin	AAD21085.1	1820	37
	Protein NDRG1	XP_418430.2	1436	4
	Crk-like protein	XP_415233.1	1067	33
	Mitogen-activated protein kinase kinase 6	XP_003642396.1	1055	33
	Histamine N-methyltransferase like	XP_001234740.1	446	18
**Gel Bands 2**	Phosphoserine phosphatase	NP_001239201.1	9344	73
	Rho GDP-dissociation inhibitor 2	XP_416182.1	9289	70
	Hypothetical protein RCJMB04 1d23	CAG30962.1	4593	43
	Ribosyldihydronicotinamide dehydrogenase	XP_418973.1	1907	50
	Sepiapterin reductase	XP_423038.1	1261	44

* The list of identified proteins in the gel filtration fraction shows only the thirteen highest scoring proteins of 93 identified.

For each protein source, identified proteins were listed according to their score calculated by ProteinLynx Global Server software (PLGS). For each protein, the sequence coverage is also indicated. Occasional peptide hits corresponding to keratins have not been included in the table.

PLGS Score is calculated by the Protein Lynx Global Server (v2.4) software using a Monte Carlo algorithm to analyze all acquired mass spectral data and is a statistical measure of accuracy of assignation. A higher score implies a greater confidence of protein identity.

### Analysis of the HNMT-like Protein Sequence

**Figure 4:**
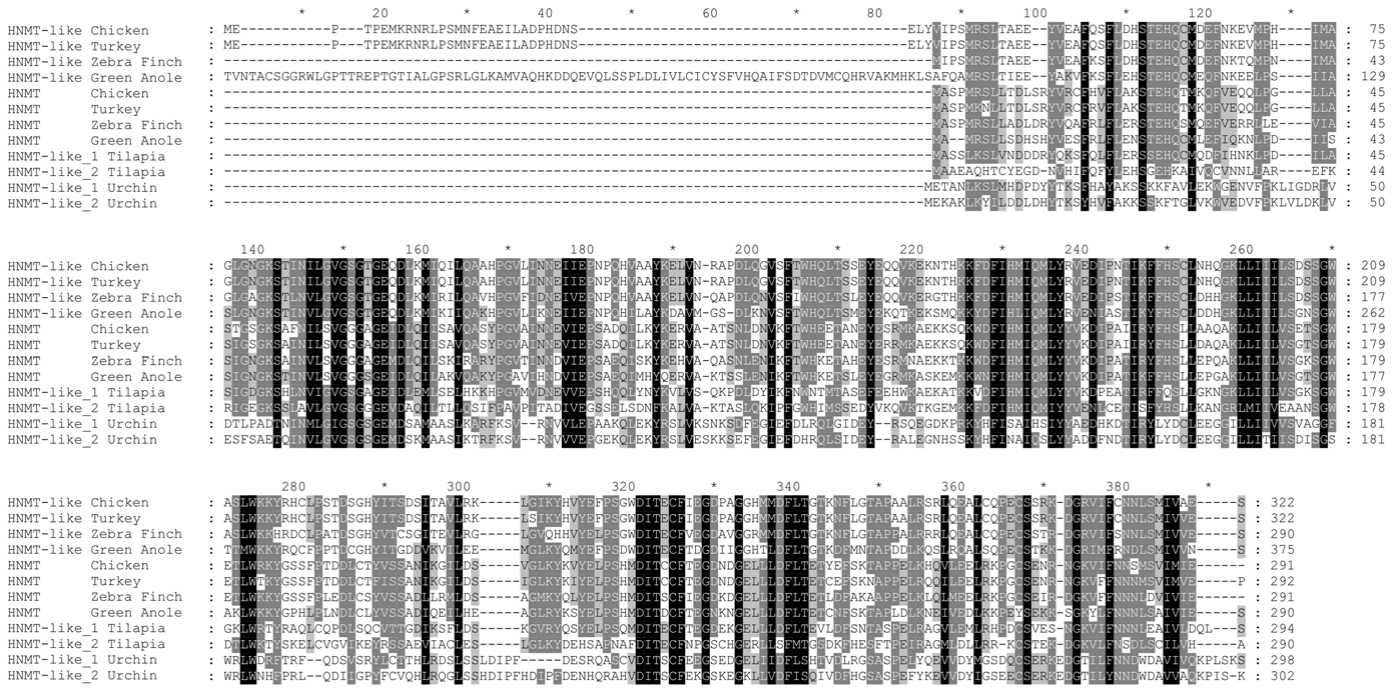
Amino acid sequence alignment of selected HNMT-like proteins with paralogue HNMT proteins. Sequences of turkey HNMT-like protein (GenBank Accession Number: XP_003207778.1), Zebra Finch HNMT-like protein (XP_002194298.1), the Green Anole HNMT-like protein (XP_003215153.1), the Nile Tilapia HNMT-like_1 and HNMT-like_2 proteins (XP_003445374.1 and XP_003445450.1, respectively), and the Sea Urchin HNMT-like_1 and HNMT-like_2 proteins (XP_786900.1 and XP_792696.1, respectively) were identified by Protein Blast searches with the use of chicken HNMT-like protein sequence (XP_001234740.1) and aligned with HNMT sequences of chicken (GenBank Accession Number: XP_422143.2), turkey (XP_003207782.1), Zebra Finch (XP_002194327.1) and the Green Anole (XP_003215142.1) using M-Coffee [27]. Level of residues conservation is indicated by black (100%), dark grey (70% and more) and light gray (50% and more) background.

As shown in the sequence alignment presented in Fig. 3, the chicken HNMT-like protein (GenBank accession number XP_001234740.1) shows 49% identity with chicken histamine N-methyltransferase (HNMT). Surprisingly, HNMT-like protein shares a lower sequence identity with chicken HNMT than the latter enzyme with its human orthologue (60%). Furthermore, amino-acid substitutions in HNMT-like protein are also detected in motifs that are strictly conserved in HNMT (cf. Fig. 3 and Fig. 4). In particular, Tyr147 and Cys196 interacting with the amino group of histamine in the active site of HNMT [19] are replaced by Arg177 and Gly226 in HNMT-like protein indicating that histamine binding to the active site might be disturbed in HNMT-like protein. Taking into account that the protein is encoded by the gen *hnmt-like* present on chromosome 7, located in the direct neighborhood of *hnmt* gene, it is likely that *hnmt-like* might be a result of gene duplication event, and the protein might acquire a new enzymatic activity.

Protein BLAST [20] searches indicated the presence of sequences very closely related to chicken HNMT-like protein in other birds (*Meleagris gallopavo* and *Taeniopygia guttata*, GenBank accession numbers: XP_003207778.1 and XP_002194298, respectively) and in a model reptile – the green anole lizard (*Anolis carolinensis*, GenBank accession number XP_003215153.1). HNMT-like proteins appear to be larger than their paralogues, revealing the presence of additional amino-acids at their N-terminus (about 30 and 100 residues in chicken and lizard HNMT-like proteins, respectively, cf. Fig. 4). BLAST searches did not detect any similarity between N-terminus of chicken HNMT-like protein and all other currently available sequences of vertebrate proteins. Intriguingly, N-terminus of the lizard HNMT-like protein shares nearly 100% identity with lizard dapper homolog 1-like protein (GenBank accession number XP_003227188.1) indicating that in fact, the former protein is a fusion of two different proteins.

All found *hnmt-like* genes are located in close proximity to *hnmt* gene. Two *hnmt-like* genes appear to be present in fish (e.g. *Oreochromis niloticus*, GenBank gene symbols LOC100703530 and LOC100704419) and the sea urchin (*Strongylocentrotus purpuratus*, GenBank gene symbols LOC581824 and LOC587894) - a close nonchordate relative of vertebrates. Surprisingly, four *hnmt-like* genes are neighbors on a chromosome of the another nonchordate deuterostome - Acorn Worm (Saccoglossus kowalevskii). One of fish HNMT-like proteins (GenBank accession number XP_003445374.1) shows slightly higher sequence similarity to chicken HNMT-like protein (47%) than the other fish HNMT-like protein (39%, GenBank accession number XP_003445450.1). Invertebrate HNMT-like proteins are equally similar to both vertebrate paralogues (about 26–30% sequence identity). Surprisingly, no HNMT-like protein ortologue was detected in available genomes and proteomes of amphibian and mammalian species. These findings suggest that carnosine N-methyltransferase gene originated from an ancestral *hnmt-like* that underwent a gene duplication and conversion during deuterostome evolution.

### Characterization of Chicken HNMT-like Protein

**Figure 5:**
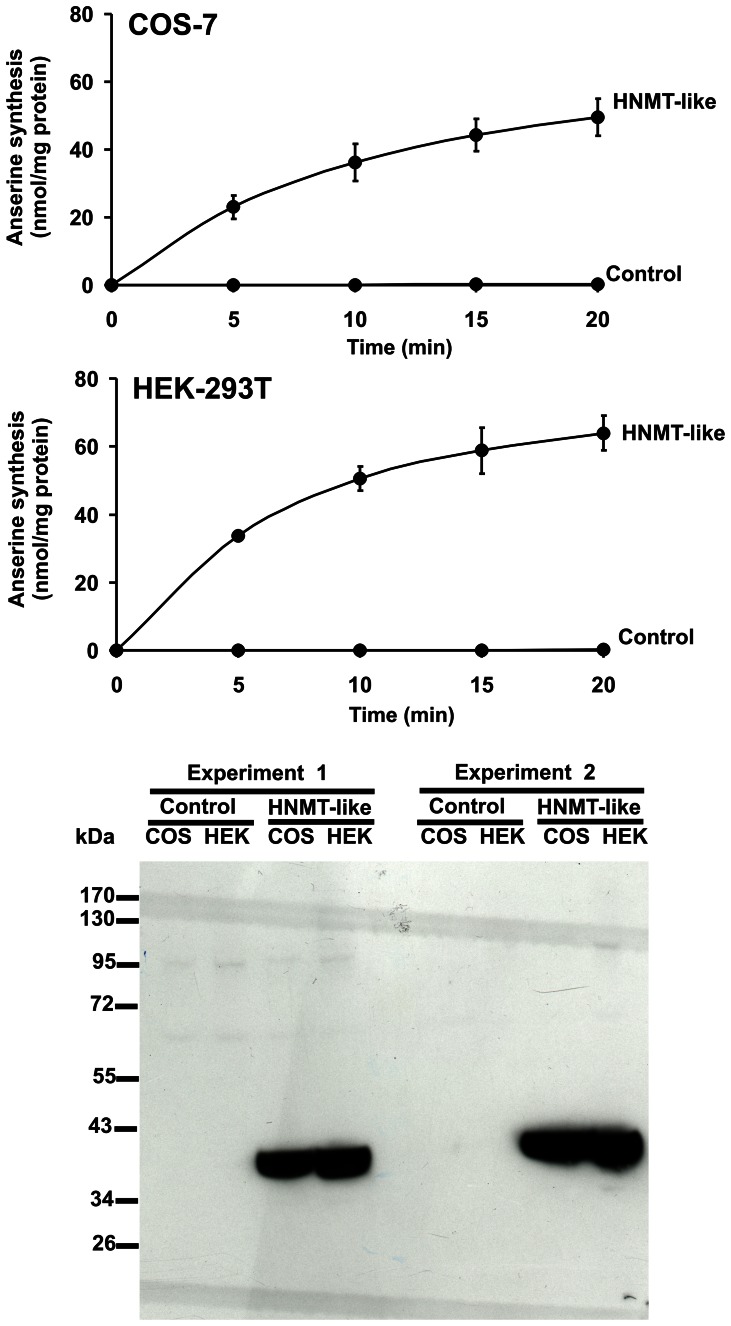
Time-course of anserine synthesis in lysates of control or HNMT-like protein-overexpressing COS-7 and HEK-293T cells. COS-7 and HEK-293T cells were transfected with either unmodified pEF6/Myc-His A vector (Control) or the same vector encoding chicken HNMT-like protein (HNMT-like) as described under “Materials and Methods”. The cell-free lysates (2–3 µg of protein) were incubated for 0, 5, 10, 15 and 20 min in the reaction mixture containing 1 µM SAM (100 pmol), as well as 440×10^3^ cpm of (^3^H)SAM. The formation of radiolabeled anserine was determined after its chromatographic separation from (^3^H)SAM. Values are the means ± S.E. of two separate transfection experiments. The presence of recombinant protein in tested lysates was verified by Western-blot analysis. Lysates (15 µg of protein) were loaded reduced onto a 10% gel, electrophoresed and blotted to nitrocellulose membrane which was then sequentially probed with a mouse primary antibody against His6 tag and a horseradish peroxidase-conjugated goat anti-mouse antibody. Secondary antibody was detected through autoradiography using chemiluminescence. COS, COS-7 cell lysate; HEK, HEK-293T cell lysate.

To confirm the molecular identity of carnosine N-methyltransferase with that of chicken HNMT-like protein, the latter one was expressed in both COS-7 and HEK-293T cells as a fusion protein with the C-terminal polyhistidine tag (Fig. 5). The recombinant enzyme catalyzed synthesis of anserine, as determined by the radiochemical assay (cf. Fig. 5). Due to ethical doubts about the use of HEK-293T cells in research [21], the protein was produced in COS-7 and purified to homogeneity (cf. Fig. 1). Gel filtration of the purified recombinant enzyme on Superdex 75 disclosed a molecular mass of ≈38 kDa for its native form (not shown), indicating that it is a monomer like the chicken enzyme.

**Figure 6:**
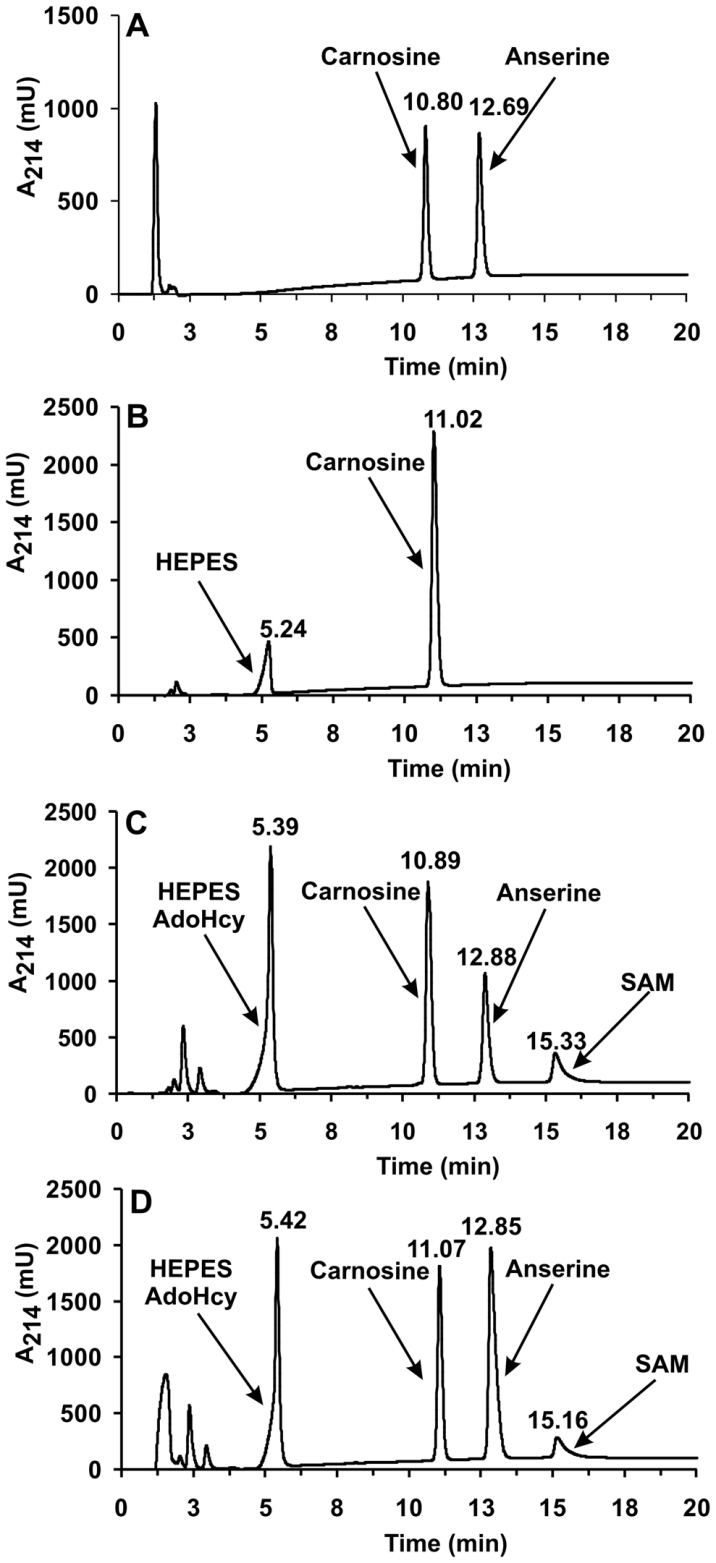
HPLC-HILIC analysis of a product formed by chicken HNMT-like protein. Chromatograms of standard mixture of carnosine and anserine (10 nmol) (A), of deproteinized reaction mixtures obtained from incubation of homogenous recombinant chicken HNMT-like protein for 12 h with 3 mM carnosine in the absence (B) or presence of 2 mM SAM (C) and following the supplementation of the former deproteinized reaction mixture with 20 nmol of anserine standard (D). The identity of all indicated compounds was confirmed by mass spectrometry. The sample processing and chromatographic conditions are described under “Materials and Methods”. AdoHcy, S-Adenosyl-L-homocysteine.

**Figure 7:**
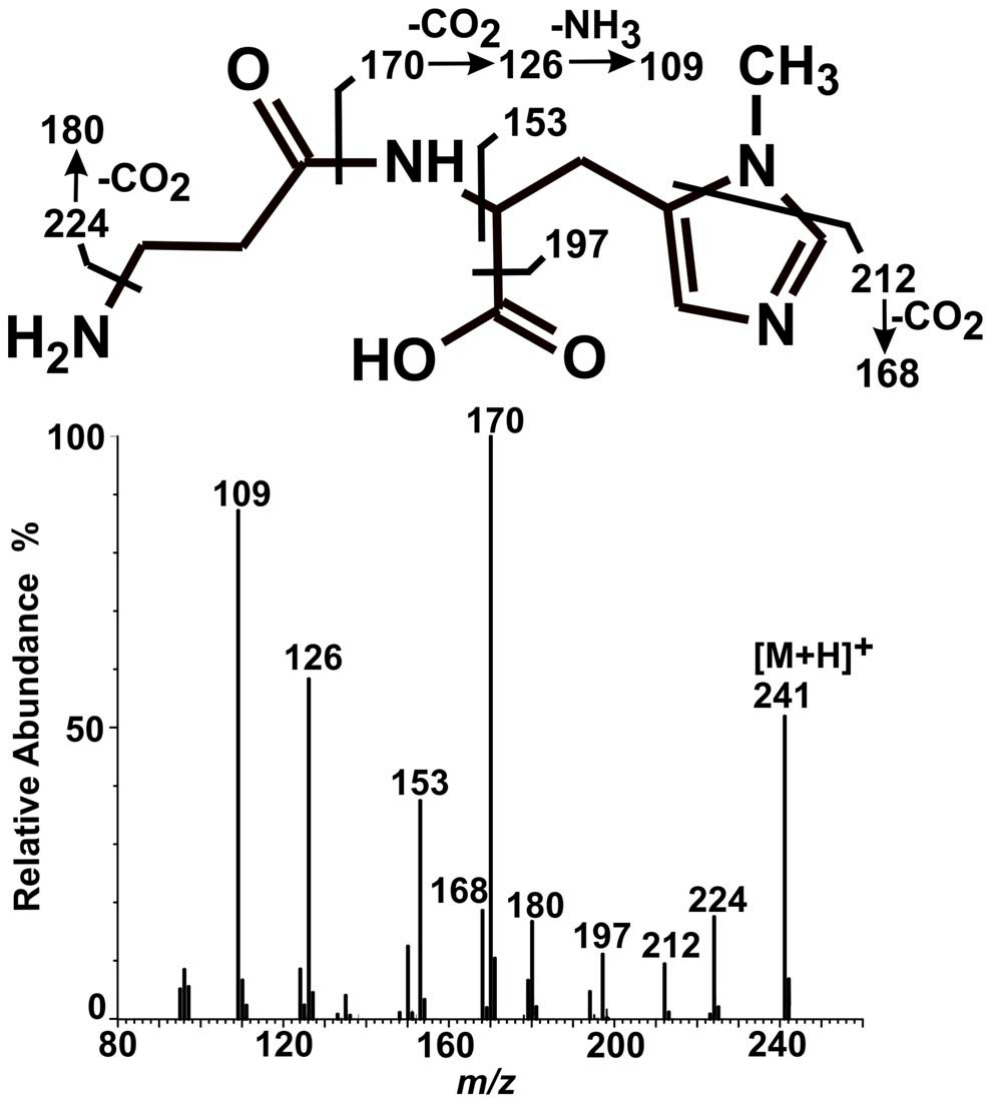
Mass spectrum of a product formed by chicken HNMT-like protein. Homogenous recombinant chicken HNMT-like protein was incubated for 12 h with 3 mM carnosine in the absence or presence of 2 mM SAM. The produced methylated dipeptide was separated from the substrates and analyzed by mass spectrometry. Both mass spectra, covering the mass range *m/z* 50–600, and tandem mass spectra (Q-TOF) for anserine precursor ion (*m/z* 241) were acquired.

The identity of the methylated product formed from carnosine by HNMT-like protein was verified by means of HPLC – HILIC MS/MS. As shown in Fig. 6, chromatographic analysis of the generated product revealed its co-migration with a commercial anserine standard. The addition of anserine to the reaction mixture resulted in a clear and selective increase in the peak area of the product (from 174 to 533 mAU × min) disclosing its identity as anserine (cf. Fig. 6). No anserine signal was detected in the control reaction with no SAM in the medium. Analysis of the product by electrospray mass spectrometry indicated the presence of a protonated molecular ion with *m/z* 241, as expected for anserine (not shown). As shown in Fig. 7, tandem mass spectrometry analysis of this ion revealed a fragmentation pattern in agreement with the anserine structure which was indeed identical with that of commercial anserine (not shown). Detected fragmentation spectrum of anserine was also in agreement with the available fragmentation spectrum of this dipeptide (MassBank Record: PR100392).

The specificity and kinetic properties of the native and recombinant chicken carnosine N-methyltransferases are shown in Tables 3 and 4. Several compounds structurally related to carnosine were tested as possible methyl group acceptors. No significant activity was detected with all tested compounds added at 10- (cf. Table 3), 1- , 0.1- and 0.01 mM concentrations (not shown), and carnosine was the only substrate for the enzyme. In agreement with reported data [22] histamine at 10- and 1 mM concentrations inhibited human HNMT activity (not shown). Thus, the data of Table 3 show only the values at 0.1 mM histamine concentration. Both native and recombinant enzymes have high affinity for S-adenosyl-L-methionine (11- and 14 µM, respectively) and carnosine at 1.6 mM concentration is required to detect half-maximal synthesis of anserine. Much lower *V_max_* and *k_cat_* values for the native enzyme in comparison to those determined for the recombinant one are likely due to insufficient purification of the native protein, indicating that about 3500-fold purification is required to obtain homogenous enzyme from the chicken pectoral muscle.

**Table 3:** (^3^H)methylation of various imidazole group-containing compounds catalyzed by carnosine N-methyltransferase.

Methyl-group acceptor	Carnosine N-methyltransferase (nmol min^−1^ mg^−1^)	HNMT-like protein (nmol min^−1^ mg^−1^)
Carnosine	11.40±0.71	49.00±1.22
Anserine	0.07±0.07	0.31±0.12
Imidazole-4-acetic acid	0.26±0.67	0.30±0.44
Imidazole-4-acrylic acid	0.10±0.84	0.95±0.24
L-Histidine	0.03±0.09	0.06±0.16
Histamine	0.21±0.03	0.09±0.02
Imidazole	0.17±0.01	0.26±0.17

The transfer of methyl group from SAM was determined with the use of homogenous recombinant chicken histamine N-methyltransferase-like protein (HNMT-like) and chicken muscle carnosine N-methyltransferase purified by chromatography on DEAE-Sepharose, Q-Sepharose, Superdex 200 and Superdex 75. Enzyme preparations were incubated for 10–15 min in the presence of 1 µM (^1^H+^3^H)SAM and 10 mM of the indicated methyl-group acceptor with the exception of histamine that was added at 0.1 mM concentration. Values are the means ± S.E. of two or three separate experiments.

**Table 4:** Kinetic properties of chicken carnosine N-methyltransferase.

	Carnosine N-methyltransferase			HNMT-like protein		
Substrate	V_max_ (nmol min^−1^ mg^−1^)	K_m_ (mM)	k_cat_ (s^−1^)	V_max_ (nmol min^−1^ mg^−1^)	K_m_ (mM)	k_cat_ (s^−1^)
Carnosine	13.82±0.37	1.646±0.157	0.008±0.000	51.87±0.64	1.628±0.071	0.034±0.001
S-adenosyl-L-methionine	153.50±3.75	0.011±0.001	0.093±0.002	786.0±19.86	0.014±0.001	0.519±0.013

Kinetic properties were determined with the use of homogenous recombinant chicken HNMT-like protein and chicken muscle carnosine N-methyltransferase purified by chromatography on DEAE-Sepharose, Q-Sepharose, Superdex 200 and Superdex 75.

Determinations for S-adenosyl-L-methionine (SAM) were performed with enzyme preparations that were incubated for 10–15 min in the presence of 20 mM carnosine and variable concentrations of (^1^H+^3^H)SAM, while the measurements for carnosine were done in the presence of non-saturating 1 µM concentration of (^1^H+^3^H)SAM. Values are the means of three separate experiments. The S.E. value is also given.

## Discussion

### Molecular Identification of Carnosine N-Methyltransferase

Carnosine and anserine have long been a subject of research, but biochemical properties and molecular identity of enzymes that catalyze their synthesis have remained unknown. Only recently, ATP-grasp domain-containing protein 1 has been identified as carnosine synthase, explaining the biosynthesis of carnosine [9]. The present work reports the identification of carnosine N-methyltransferase as chicken HNMT-like protein, disclosing the identity of anserine-producing enzyme. This conclusion results from the following findings: 1) chicken pectoral muscle is a very rich source of carnosine N-methyltransferase [10] and HNMT-like protein was the only methyltransferase identified in the most highly purified preparation from the chicken muscle, 2) the recombinant HNMT-like protein catalyzes the transfer of methyl group from SAM onto carnosine, yielding anserine and 3) the identity of the product made by the recombinant enzyme was confirmed by both hydrophilic interaction chromatography and hybrid mass spectrometry.

HNMT-like was just one of numerous proteins identified in the most purified chicken muscle preparation indicating that the enzyme might form a complex with other protein(s). Since the homogeneously purified recombinant HNMT-like protein was found to catalyze synthesis of anserine, it is unlikely that the presence of other protein(s) in the reaction mixture is required for its activity. In our opinion, these co-purified proteins are contaminants resulting from incomplete purification process. Taking into account that the proteins were separated by size using two different Superdex columns, the surprising presence of high molecular weight proteins (form 50 kDa to 95 kDa, cf. Fig. 2) in the enzyme preparation might be due to their adsorption to the gel matrix in experimental conditions applied [23]. However, transient interactions of the enzyme with co-purifying proteins cannot be absolutely excluded.

Molecular weight of the native chicken carnosine N-methyltransferase differed from the weight estimated for the recombinant HNMT-like protein (45 kDa and 36 kDa without His6-tag, respectively). This overestimation of the native enzyme weight is probably due to the fact that Superdex 200 provides clearly lower selectivity and resolution of the separation process for proteins in the molecular weight range from about 10 to 60 kDa, compared with Superdex 75 [24].

### Kinetic Properties and Substrate Specificity of Carnosine N-Methyltransferase

Our results show that both the native enzyme and its recombinant form exhibit similar kinetic parameters, confirming the correct identification of the enzyme present in the purified muscle preparation and indicating a much higher affinity of the methyltransferase for SAM than for carnosine. The latter observation is in general agreement with data reported by MacManus [10] who, in the presence of 8-fold purified chicken muscle preparation, reported *K_m_* values equal to 0.09 mM and 4 mM for SAM and carnosine, respectively. Furthermore, the specificity studies performed here indicate that the chicken enzyme utilizes exclusively carnosine, showing similar properties to that described previously [10]. We believe that although HNMT and HNMT-like proteins share almost 50% sequence identity, histamine is not a substrate for carnosine N-methyltransferase at least due to the substitution of amino acid residues that are important for the correct binding of the amine. This conclusion is consistent with the suggestion that the inhibitory action of iodacetamide on HNMT activity results from the alkylation of Cys198 that interacts with amine group of histamine in the active center of the transferase [19].

### Phylogeny

The *hnmt* and *hnmt-like* genes evolved from pre-existing genes that arose through the duplication of an ancestral gene. Duplication event happened most likely at early stages of the deuterostome evolution, since *hnmt-like* genes tandems are present on the chromosomes of species belonging to three major phyla of the Deuterostomia: Chordata, Hemichordata and Echinodermata. No orthologues of *hnmt-like* genes are detected in genomes of the protostomes. The presence of the same highly conserved motifs in sequences of both invertebrate and vertebrate proteins may indicate that invertebrate HNMT-like proteins are also SAM-dependent methyltransferases.

It is intriguing that amphibian and mammalian genomes that are available do not contain orthologues of carnosine N-methyltransferase gene (*hnmt-like*). Anserine was not detected in tissues of frogs [3], confirming these observation. However, taking into account a vast shortage of amphibian genomic data, one cannot exclude that some amphibian have *hnmt-like* gene and are able to produce anserine. In the contrary, both anserine and carnosine N-methyltransferase activities have been reported in some mammals [3]. Raghavan and coworkers [11] reported a partial purification of carnosine N-methyltransferase from rabbit muscle, occurring to be an 85 kDa protein. Indeed, preliminary data from our laboratory indicate the presence of only one form of carnosine-methylating enzyme in rat muscle which exhibits molecular weight equal to 94 kDa, as estimated by the gel filtration. We believe that another anserine-producing enzyme is present in mammals and it might be a product of convergent evolution which commonly happens among methyltransferases [25].

## Conclusions

In the current work, we have identified carnosine N-methyltransferase that catalyzes the last step in the anserine synthesis pathway. The enzyme is closely related to histamine N-methyltransferase and appears to be present in a majority of anserine-producing species. The lack of the identified gene in mammalian genomes suggests that another form of carnosine N-methyltransferase may exist in these vertebrate species.
